# The Relationship between the Sense of Coherence of Dental Hygiene Students in Their Graduation Year and Their View of the Profession and Attitude to Work: A Cross-Sectional Survey in Japan

**DOI:** 10.3390/ijerph17249594

**Published:** 2020-12-21

**Authors:** Rumi Tano, Hiroko Miura, Katsuo Oshima, Kanako Noritake, Hideki Fukuda

**Affiliations:** 1 National Institute of Public Health, Wako, Saitama 351-0197, Japan; fukuda.h.aa@niph.go.jp; 2 School of Dentistry, Health Sciences University of Hokkaido, Ishikari-gun, Hokkaido 061-0293, Japan; hmiura@hoku-iryo-u.ac.jp; 3 The Nippon Dental University College at Tokyo, Chiyoda-ku, Tokyo 102-8159, Japan; oshima@tky.ndu.ac.jp; 4 Oral Diagnosis and General Dentistry, University Hospital of Dentistry, Tokyo Medical and Dental University (TMDU), Bunkyo-ku, Tokyo 113-8510, Japan; noritake.irm@tmd.ac.jp

**Keywords:** sense of coherence, view of occupations, view of career, dental hygiene students, exhaustive survey

## Abstract

Objective: The need to make sense of coherence in placement support for student dental hygienists has been shown. On this basis, this study investigated the relationship between the sense of coherence of student dental hygienists and their view of the profession and attitude to work in order to clarify how they perceive their prospects for employment. Methods: The subjects were graduation-year students at all of the dental hygienist training institutions in Japan, and anonymous, self-administered questionnaires were sent to the institutions by post in 2019. The results were analyzed by χ^2^ tests, as well as one-way analysis of variance and multiple comparisons using Tukey’s test, with the level of significance set at 5%. Results: Of 6270 questionnaires that were returned, 6264 were analyzed. The sense of coherence (SOC) component senses were manageability (F(26,221) = 5306.06, *p* < 0.01), meaningfulness (F(26,222) = 4373.48, *p* < 0.01), and comprehensibility (F(26,216) = 3986.12, *p* < 0.01), with meaningfulness scoring significantly higher than the other two (*p* < 0.01). Analysis with SOC scores divided into the low, medium, and high groups showed a relationship between the SOC of student dental hygienists and their view of the profession and attitude to work (*p* < 0.01), such that higher SOC scores were associated with a better view of the profession and a better attitude to work (F(26,225) = 282.18, *p* < 0.01). Conclusions: The results suggest that education that increases SOC in dental hygienist training programs may positively affect future prospects for student dental hygienists.

## 1. Introduction

Sense of coherence (SOC) is the core concept of Antonovsky’s idea of salutogenesis [1], and it refers to the ability of an individual or group to realize their growth-enhancing potential by coping with stressors [2]. SOC is defined as “The sense of coherence is a global orientation that expresses the extent to which one has a pervasive, enduring though dynamic feeling of confidence that (1) the stimuli deriving from one’s internal and external environments in the course of living are structured, predictable, and explicable; (2) the resources are available to one to meet the demands posed by three stimuli; and (3) these demands are challenges, worthy of investment and engagement”. It comprises the three-component senses of manageability, meaningfulness, and comprehensibility [1]. The concept of SOC expresses the way we look at, face up to, and relate to the world in which we live, and it may be regarded as the stress coping skills or health maintenance skills that enable us to maintain physical and mental health by viewing stress as something that nurtures our growth and development and coping with it properly [3,4]. The SOC has attracted attention in a wide range of fields, including medicine, health, and education, and the SOC scale has come to be widely used around the world. Higher scores on the SOC scale indicate higher stress coping skills or health maintenance skills, and the SOC scale has been used as an index for the outcome of intervention programs [5,6]. Many prior studies have shown that SOC is a predictor of health and quality of life and acts as a buffer against stressors [7,8]. In students, it has been shown that SOC is related to awareness of stress and has a stressor-buffering effect [9,10], and that students with high SOC have better self-rated health than students with a lower SOC [11].

Similarly, the SOC scale has been used in Japan to focus on stress coping skills and health maintenance in dentistry, and the SOC of student dental hygienists has been found to correlate with stress coping skills, state/trait anxiety, and self-rated health during practical training [12,13]. A negative correlation was found between SOC and mental health status in a study of trainee dentists, showing that trainee dentists with a higher SOC had better mental health [14]. In recent years, a correlation has been reported between SOC and essential skills for working adults, indicating the need to take SOC into account when supporting student dental hygienists searching for employment [15].

It is known from prior studies that dental hygiene students chose the profession to work with and help people and have flexible work schedules [16]. Also, dental hygiene students also agreed with receiving the education to increase the capacity to work with underserved groups and expanded clinical abilities for entering the profession of dental hygiene [17]. A tendency has been identified in Japan for dental hygienists to leave the profession at an early stage [18], and it is therefore likely that investigating the type of effects that SOC has for student dental hygienists, as well as how the relationship with their view of the profession and their attitudes to work varies according to the strength of SOC, will contribute to any examination of dental hygienist training. In particular, the discussion focused on SOC based on the situation of student dental hygienists nearing completion of their studies may give some suggestions for investigation of career education.

The purpose of this study was to clarify how SOC relates to views of the dental hygienist profession and attitude to work in Japanese students in the graduation year of dental hygienist training courses.

## 2. Materials and Methods

An anonymous, self-administered survey of graduation-year students at all Japanese dental hygienist training institutions was conducted by post over 3 weeks in November 2019. A total of 162 training institutions were surveyed, comprising 153 (94.4%) vocational colleges with 3-year programs and 9 (5.6%) universities with 4-year programs. A corresponding number of questionnaire forms was sent to each institution with a request to distribute the forms and collect them when completed.

The questionnaire items were as follows: (1) personal attributes (sex, age); (2) number of years the respondent wishes to work as a dental hygienist at the first place of employment after graduation; (3) whether the respondent is still happy with the choice to become a dental hygienist; (4) whether the respondent considers being a dental hygienist to be a worthwhile job; (5) whether the respondent wishes to be a dental hygienist for all their working life; (6) experience of receiving career education at the dental hygienist training institution; (7) career prospects; (8) desire to continue participation in training courses related to dentistry; (9) intention to obtain certification as a dental hygienist; and (10) SOC scale score.

The SOC scale used was the 7-point short version SOC3 (SOC 3-UTHS; University of Tokyo Health Sociology version of SOC 3 scale) [19], comprising the three items of manageability (“I am able to find solutions to the hardships and problems that occur every day”), meaningfulness (“I think it is worth facing and dealing with some of the hardships and problems of life”), and comprehensibility (“I am able to understand and predict the hardships and problems that occur every day”). Respondents gave their answers on a 7-point scale from 1 (strongly agree) to 7 (strongly disagree).

For the statistical analysis, χ^2^ test and one-way analysis of variance (ANOVA), were carried out using SPSS Statistics Ver. 25.0 (IBM Japan), with the significance level set at 5%. In the analysis, all SOC scores were reversed and then scored according to the number of points. Since no benchmarks for high or low SOC scale scores and no cutoff point were established [1], the survey results were classified into tertiles about the 33rd and 67th percentiles, with the three resulting groups taken as the low score group (3–12 points), the medium score group (13–16 points), and the high score group (17–21 points). The questions regarding the view of the profession and attitude to work were scored as follows: number of years the respondent wishes to work as a dental hygienist at the first place of employment after graduation scored 1 for ≥3 years; whether the respondent is still happy with the choice to become a dental hygienist, whether the respondent wishes to continue working as a dental hygienist, and whether the respondent considers being a dental hygienist to be a worthwhile job all scored 1 for “Yes”; career prospects are “mapped out” or “somewhat mapped out” scored 1; the desire to continue participating in training courses and intention to obtain certification as a dental hygienist scored 1 for “strongly agree” and “somewhat agree”; and experience of receiving career education at the dental hygienist training institution scored 1 for “Yes”. All other responses scored 0, giving a maximum possible score of 8. The three component senses were compared by analysis of variance of the mean scores, and multiple comparisons were carried out using Tukey’s test. Also, the mean scores for the view of the profession and attitude to work and the mean scores in the three SOC groups were compared by analysis of variance, and multiple comparisons were carried out using Tukey’s test. The relationships between variables relating to view of the profession and attitude to work and SOC score group were investigated using a χ^2^ test.

This study was carried out with the cooperation of the Japan Association for Dental Hygienist Education and approved following an ethical review by the National Institute of Public Health (approval no. NIPH-IBRA#12254). A collection envelope was attached to the questionnaire form, and the students themselves sealed their completed questionnaires in the envelopes, which were then returned together by the institution.

## 3. Results

A total of 162 training institutions were surveyed, of which 6270 were returned. Excluding 6 that were not filled in, 6264 questionnaires were analyzed. A total of 141 training institutions were returned, comprising 134 (95.0%) vocational colleges with 3-year programs and 7 (5.0%) universities with 4-year programs. Of these, 6177 (98.6%) were women; the respondents were aged from 20 to 64 years, with a mean age (standard deviation) of 21.7 (3.5) years. By age group, 5956 (95.1%) were in their 20s, 214 (3.4%) in their 30s, and 46 (0.7%) in their 40s.

First, mean scores in the high, medium, and low SOC score groups were compared by analysis of variance with the three-component senses as dependent variables in order to determine whether the three-component senses differed according to the SOC score. The results showed significant relationships between the three-component senses and the SOC score as follows: manageability, F(26,221) = 5306.06 (*p* < 0.01); meaningfulness, F(26,222) = 4373.48 (*p* < 0.01); and comprehensibility, F(26,216) = 3986.12 (*p* < 0.01). A multiple comparison using Tukey’s test (Table 1) showed a significant difference in the mean score for the SOC component senses among all three SOC groups (*p* < 0.01).

**Figure 1:**
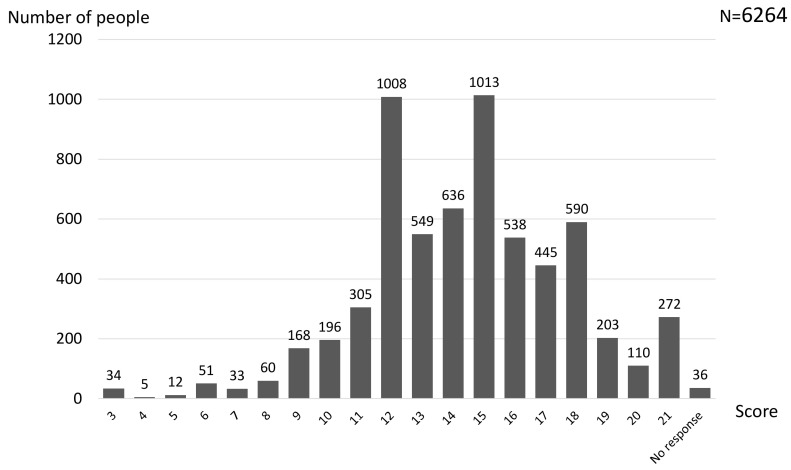
SOC scale (SOC 3-UTHS) scores of student dental hygienists. SOC 3-UTHS: University of Tokyo Health Sociology version of the sense of coherence 3 scale.

Next, the total score of the three items was calculated for each student, with the maximum score being 21. Scores were in the range 3–21, with 15 as the most common score (1013 respondents, 16.2%), followed by 12 (1008 respondents, 16.1%) (Figure 1). The mean (standard deviation) was 14.4 (3.4), and a score of 12 or more accounted for 85.7% of the total number of responses.

That the view of the profession and the attitude to work of the student dental hygienists were significantly related to SOC (*p* < 0.01) (Table 2). The results of the ANOVA of the view of the profession and attitude to work of the student dental hygienists in the low, medium, and high SOC score groups were F(26,225) = 282.18 (*p* < 0.01), showing a significant relationship with SOC score. The results of Tukey’s multiple comparisons showed significant differences in view of the profession and attitude to work among all SOC score groups, indicating relationships between the SOC score and view of the profession and attitude to work (*p* < 0.01).

## 4. Discussion

This cross-sectional, exhaustive survey used a self-administered questionnaire sent to graduation-year students at all dental hygienist training institutions in Japan to investigate how SOC relates to the view of the profession and attitude to work using statistical analysis. A total of 7216 students took the national dental hygienist examination in the surveyed academic year from April 2019 to March 2020 [20], so even allowing for fluctuations in enrollment and the number of qualified candidates, 6270 respondents may be considered a high response rate. The results of the survey show that there is a relationship between SOC in student dental hygienists and their view of the profession and attitude to work, suggesting that improving SOC is important in dental hygienist education.

In the survey, over 90% of respondents were women, and the mean (SD) SOC score was 14.4 (3.4). The survey results resemble the mean (SD) SOC, score of a representative nationwide sample of Japanese women aged 25–29 years of 14.8 (3.4) [21], so it was therefore found that the SOC of student dental hygienists in their graduation year is no different from that of women in general of a comparable age group. SOC is an essential resource for specialist professionals providing healthcare, and, as an example, it is known to be a predictive factor for burnout or occupational dissatisfaction among nurses [22]. As specialized health professionals providing interpersonal care, dental hygienists have an even greater requirement for stress coping skills and health maintenance skills than members of the general public, and there is thus a need to increase the SOC of dental hygienists.

The present study suggests that, of the three-component senses of SOC, meaningfulness scores significantly higher than manageability or comprehensibility among student dental hygienists. The factors comprising meaningfulness include the pursuit of novelty, which indicates an attitude of taking issues that are currently faced positively and constructively approaching future events, and meaningfulness is therefore considered important because it impacts upon the other two senses [1]. Meaningfulness is a concept that involves motivation. It is thought that increasing meaningfulness means that, even with low manageability and comprehensibility, a person will be interested in the problems that confront them, allowing understanding and resources to solve the problem to be obtained. There is, therefore, a need for dental hygienist training that aims to increase meaningfulness, while also emphasizing manageability and comprehensibility, in order to increase the SOC of student dental hygienists.

The present survey results suggest that the view of the profession and the attitude to work of dental hygienist students may be significantly related to SOC. Students with higher SOC wanted to work for a more significant number of years, had greater positivity about their choice to become dental hygienists, wanted to continue in the profession, and considered the job worthwhile. They also considered their career prospects to be mapped out, were more motivated toward self-improvement, and were more aware of having career education. These findings support the results of prior studies that found an association between low SOC and early withdrawal from the profession, that student nurses with higher SOC have more excellent career prospects [23], and the recognition of occupational identity among student dental hygienists is positive [24]. Also, the SOC scale has been shown to be effective for coping strategies relating to the view of their profession and for medical career development in student oral health specialists [25,26], suggesting that it might be useful as an indicator of a positive attitude toward the future in student dental hygienists.

In recent years, a successful experience at school increases career orientation [27], and that career support for student dental hygienists should take SOC into account [15]. SOC is not an innate characteristic. Preferably, it is formed later in life [1], and it would be desirable for dental hygienist education to develop and form SOC. According to Antonovsky, the shaping factors of SOC are General Resistances Resources [28], which are three types of experiences: the experience of living within a stable set of rules and a stable sphere, the experience of successfully coping with moderate stress, and the experience of participation in critical decision-making [29]. Respectively, these experiences are known as consistency, load balance, and participation in shaping outcomes [29]. It is believed that the combination and repetition of these experiences lead to the formation or strengthening of SOC. In particular, it has been pointed out that nurturing SOC in schools is important from an educational perspective [30], given that SOC contributes to task achievement in adolescence [31]. It has been reported that a factor affecting SOC in student nurses is the provision of an environment for mutual study, and an environment that provides abundant external resources and encourages their use is essential [23,32]. We therefore consider that education supporting successful coping within an appropriately balanced load experience that includes academic tasks and extracurricular activities will increase SOC in dental hygienist programs. Specifically, during the process whereby student dental hygienists acquire knowledge and skills through lectures, exercises, and practical classes, training institutions need to give support to students with low SOC by providing an environment where the students can work independently and set tasks that take the load into account. Education that increases SOC in this way may lead to a positive attitude toward future prospects of finding work as a dental hygienist and become the foundation for career aspirations for student dental hygienists.

This is the first study either in Japan or abroad to use an exhaustive survey to investigate the relationship between the SOC of student dental hygienists in their graduation year and their view of the profession and attitude to work, and it is of considerable significance in terms of its originality and generalizability. In addition, the results from the SOC scale yielded findings that may contribute to the examination of career education in dental hygienist programs. However, since this was a cross-sectional study, there are limits to the elucidation of the causal relationship between the SOC of student dental hygienists and their view of the profession and attitude to work. Also, this study was recruited from a single reality. In addition, since this study does not account for different backgrounds and curriculums existing in other countries, the generalizability of this study is limited to Japan. It is hoped that the effects of SOC on students’ views of the profession and attitude to work will be investigated through future research by interventional studies and follow-up surveys.

## 5. Conclusions

The results of the present study showed that the SOC score was significantly related to the view of the profession and the attitude to work among dental hygienist students. Students with higher SOC score responded that they wanted to work for more years (*p* < 0.01), had greater positivity about their choice to become dental hygienists (*p* < 0.01), wanted to continue in the profession (*p* < 0.01), and considered their job as worthwhile (*p* < 0.01). They also considered their career prospects to be mapped out (*p* < 0.01), were more motivated toward self-improvement (*p* < 0.01) and were more aware of having career education (*p* < 0.01). Undergraduate education that improves SOC scores may lead to a more positive attitude toward the prospect of employment as a dental hygienist and may form the basis of career aspirations.

## Figures and Tables

**Table 1:** Relationships between the SOC score and component senses in each SOC group.

	Total	Mean (Standard Deviation)			Multiple Comparison Tukey’s Test		
SOC		SOCscore					
Component Sense		Low Group 3–12	Medium Group 13–16	High Group 17–21			
Manageability	4.7 (1.2)	3.5 (0.9)	4.7 (0.7)	6.2 (0.7)	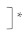		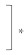
Meaningfulness	5.1 (1.2)	3.9 (1.0)	5.2 (0.8)	6.4 (0.6)		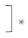	
Comprehensibility	4.6 (1.3)	3.4 (1.0)	4.6 (0.8)	6.0 (0.8)			

*: *p* < 0.01. SOC: sense of coherence.

**Table 2:** View of profession and attitude to work by SOC score group.

	*n* (%)				*p* Value (χ^2^ Test)
	Total *n* = 6228	Low Score Group (Score 3–12)	Medium Score Group (Score 13–16)	High Score Group (Score 17–21)	
		*n* = 1872	*n* = 2736	*n* = 1620	
Number of years I wish to work as a dental hygienist at the first place of employment after graduation					
<3 years	1122 (19.4)	413 (23.9)	450 (17.6)	259 (17.2)	<0.01
3–<5 years	2612 (45.1)	797 (46.2)	1190 (46.5)	625 (41.6)	
5–<10 years	1425 (24.6)	355 (20.6)	677 (26.4)	393 (26.1)	
≥10 years	629 (10.9)	160 (9.3)	243 (9.5)	226 (15.0)	
I am still happy with my wish to become a dental hygienist					
Yes	3738 (60.5)	848 (45.6)	1692 (62.3)	1198 (74.7)	<0.01
No	315 (5.1)	160 (8.6)	109 (4.0)	46 (2.9)	
Don’t know	2124 (34.4)	851 (45.8)	913 (33.6)	360 (22.4)	
I think a dental hygienist’s job is worthwhile					
Yes	5232 (84.3)	1380 (74.0)	2375 (87.0)	1477 (91.6)	<0.01
No	65 (1.0)	35 (1.9)	18 (0.7)	12 (0.7)	
Don’t know	910 (14.7)	449 (24.1)	337 (12.3)	124 (7.7)	
I want to work as a dental hygienist for all my working life					
Yes	3128 (50.4)	709 (38.0)	1420 (52.0)	999 (61.9)	<0.01
No	619 (10.0)	256 (13.7)	240 (8.8)	123 (7.6)	
Don’t know	2464 (39.7)	900 (48.3)	1073 (39.3)	491 (30.4)	
I have received career education					
Yes	1639 (26.4)	367 (19.7)	702 (25.7)	570 (35.3)	<0.01
No	870 (14.0)	244 (13.1)	378 (13.8)	248 (15.3)	
Don’t remember	3702 (59.6)	1252 (67.2)	1651 (60.5)	799 (49.4)	
Career prospects					
Mapped out	495 (8.0)	67 (3.6)	141 (5.2)	287 (17.8)	<0.01
Somewhat mapped out	2159 (34.7)	400 (21.4)	990 (36.2)	769 (47.6)	
Hardly mapped out	2754 (44.3)	947 (50.8)	1334 (48.8)	473 (29.3)	
Not mapped out	806 (13.0)	452 (24.2)	269 (9.8)	85 (5.3)	
I want to continue participating in training courses					
Strongly agree	1029 (16.6)	166 (8.9)	416 (15.2)	447 (27.7)	<0.01
Somewhat agree	3138 (50.5)	848 (45.4)	1457 (53.3)	833 (51.6)	
Somewhat disagree	1620 (26.1)	630 (33.7)	731 (26.7)	259 (16.0)	
Strongly disagree	427 (6.9)	223 (11.9)	129 (4.7)	75 (4.6)	
I want to obtain certification as a dental hygienist					
Strongly agree	819 (13.2)	147 (7.9)	334 (12.2)	338 (21.0)	<0.01
Somewhat agree	2262 (36.4)	546 (29.2)	1053 (38.5)	663 (41.1)	
Somewhat disagree	1960 (31.6)	684 (36.6)	868 (31.8)	408 (25.3)	
Strongly disagree	449 (7.2)	213 (11.4)	167 (6.1)	69 (4.3)	
Don’t know about certification as a dental hygienist	722 (11.6)	277 (14.8)	310 (11.3)	135 (8.4)	

Returned questionnaires with no responses excluded. SOC: sense of coherence.
